# High Incidence and Levels of Ochratoxin A in Wines Sourced from the United States

**DOI:** 10.3390/toxins10010001

**Published:** 2017-12-21

**Authors:** Christopher Lawrence De Jesus, Amanda Bartley, Aaron Z. Welch, John P. Berry

**Affiliations:** 1 Department of Chemistry and Biochemistry, Florida International University, 3000 NE 151st Street, North Miami, FL 33181, USA; cd2975@cumc.columbia.edu (C.L.D.J.); abart043@fiu.edu (A.B.); 2 Biomolecular Sciences Institute, Florida International University, Miami, FL 33181, USA; aawelch@fiu.edu

**Keywords:** ochratoxin A, wine, United States, HPLC-FD, liquid/liquid extraction (LLE)

## Abstract

Ochratoxin A (OTA) is one of the most prevalent mycotoxin contaminants of food crops. Among the agricultural products consequently contaminated by OTA is wine. In the present study, a sample of wines sourced from the United States was assessed for OTA. Wines were primarily analyzed by high-performance liquid chromatography with fluorescence detection (HPLC-FD) coupled to a liquid-liquid extraction (LLE) technique which was developed and validated as a simplified sample preparation approach. More than 85% of the wines evaluated were found to contain OTA, at levels above the limit-of-detection (LOD = 0.1 µg L^−1^), and 76% were above the limit-of-quantitation (LOQ = 0.3 µg L^−1^) for the LLE/HPLC-FD method. More than two-thirds of the wines above the LOQ were found to exceed 1 µg L^−1^. Complementary analysis by HPLC coupled to tandem mass spectrometry (HPLC-MS/MS) confirmed OTA in 74% of the OTA-positive wines (i.e., >LOQ by HPLC-FD). Overall, both the occurrence and measured levels of OTA were generally high, specifically relative to previous assessments of OTA in wine, and two of the wines were above the only current (European Union) regulatory limit of two parts-per-billion (ppb, ~2 µg L^−1^). Possible trends with respect to geographical region and/or growing climate are noted. As the first assessment of U.S. wines in more than a decade, the overall high occurrence and levels of OTA in wine, and possible geographic and climatic trends, point to a need for regular surveillance of wines, as well as investigation of the relevant contributors to OTA occurrence toward mitigating contamination and exposure risks.

## 1. Introduction

Ochratoxins are produced by several species of the fungal genera *Aspergillus* and *Penicillium*, and are among the most widely reported of the nearly 400 recognized mycotoxin contaminants of agricultural products [1,2]. Of the known variants, the most prevalent congener, ochratoxin A (OTA), has been shown to be potently toxic in various systems [3,4]. Toxicity includes nephrotoxicity, and associated pathologies (i.e., nephropathy), in several mammalian and non-mammalian systems, as well as both genotoxic and non-genotoxic carcinogenicity [3,5]. Epidemiologically, OTA contamination of various crops has been linked, for example, to a geographically specific form of tubulointerstitial nephrites, namely *Balkan Endemic Nephropathy*, in areas of Southeastern Europe, and in particular, the agriculturally fertile Danube River Basin [5]. Based on the links between OTA and human health effects, regulatory limits have been established, particularly within the European Union (EU), for several agricultural products with respect to this toxin [6].

Among the agricultural products associated with OTA contamination, and of possible human health concern, is wine. Wine as a source of OTA is highlighted by considerable, and continuing, global increases in wine consumption [7,8]. As the leading consumer of wine, the United States (U.S.) has, in particular, seen consumption steadily rise with incremental increases—every year between 1996 and 2016—by more than 55% over the last two decades, or from 1.89 to 2.94 gallons per person per year [9]. Presence of OTA in wine occurs via fungal contamination of grapes, and subsequently *must* (i.e., crushed grapes including juice and, in the case of red wines, skins) used to make wine [10,11]. It has, in turn, been shown that OTA is not entirely removed during fermentation, and can be consequently found in the final product [10,11]. Accordingly, the EU has established limits on the levels of OTA for wine, and these current regulatory guidelines specifically prohibit wines with levels of OTA above two parts-per-billion (ppb), i.e., ~2 µg L^−1^. No such regulatory limits, however, have been currently established for OTA in wine in the U.S., or other parts of the world.

Although numerous prior studies have investigated OTA in wine [12,13,14,15,16,17,18,19,20,21,22,23,24,25,26,27,28,29,30,31,32,33,34,35,36,37,38], very limited studies [14,16,19], and none in well over a decade have investigated OTA in U.S. wines. That said, several lines of evidence suggest significant impacts of climate change and related factors, on a decadal scale, with respect to the prevalence (i.e., increases) of OTA in agricultural products including wine [39], and regular surveys will, therefore, be essential to mitigating risk exposure to OTA and other mycotoxins.

Alongside emerging concerns regarding OTA as a toxic contaminant, analytical techniques to detect and/or quantify OTA in agricultural products have evolved. Of these, sensitive detection and quantitation by high-performance liquid chromatography (HPLC) coupled to fluorescence detection (HPLC-FD)—specifically taking advantage of the inherent fluorophore of OTA—has been the most frequently utilized, and repeatedly validated [40]. When coupled to sufficient sample preparation, HPLC-FD is capable of detecting and measuring OTA in wine well below the established EU guideline (i.e., 2 µg L^−1^), and typically, in the parts-per-trillion range, e.g., ≥0.01 µg L^−1^ [40]. Although HPLC-FD has been, by far the most commonly employed analytical method, alternative approaches based, in particular, on mass spectrometry coupled to HPLC have been, likewise, developed for OTA in wine, and a range of relevant matrices [41,42], and specifically enable confirmatory identification of OTA by means of highly selective tandem mass spectrometry (i.e., HPLC-MS/MS).

Methods for extraction of OTA from diverse biological matrices, as well as subsequent sample preparation, have simultaneously evolved. The extraction/sample preparation step is, in fact, cited as a major bottleneck in the analysis of mycotoxins [43]. Notable advances include solid-phase extraction (SPE) and, in particular, commercially available (although potentially cost prohibitive) immunoaffinity columns (IAC) for highly selective cleanup and enrichment of OTA prior to analysis [44,45]. However, a growing trend with respect to the extraction and sample preparation steps has been targeting of so-called “quick, easy, cheap, effective, rugged and safe” (QuEChERS) and other simplified, and streamlined, methods [45]. Numerous studies have, indeed, reported the development and application of such techniques including very simple “dilute and shoot,” and other approaches that require little or no sample preparation [38,41,46,47].

In the present study, we developed and validated a simple and rapid liquid/liquid extraction (LLE) technique coupled to HPLC-FD, and subsequently evaluated a representative sample of wines from domestic U.S. wineries. Analysis was complemented by HPLC-MS/MS as a confirmatory technique. Results of this study—as the first survey of U.S. wines in more than a decade—were, in turn, evaluated relative to current regulatory guidelines, and compared to previous assessments of both U.S. and internationally sourced wines specifically with respect to the proposed contributions of geographic variability, including possible contributions of growing climate, to the occurrence of OTA in wine.

## 2. Results and Discussion

### 2.1. Analytical Performance of HPLC-FD Coupled to LLE

Toward the development of a simplified approach to analysis of OTA in wine, the present study adapted LLE to a previously developed HPLC-FD analytical technique [48], and subsequently applied this methodology to the survey of OTA in U.S. wines (see Section 2.2, below). Sample preparation was achieved by LLE of OTA from wine (i.e., aqueous phase) into chloroform, followed by recovery of OTA-containing organic phase (and evaporation to remove extraction solvent). This simplified approach to extraction and sample preparation effectively replaces the use of IAC, and other SPE techniques, which add to both time/labor and cost of analyses. Assessment of accuracy and precision suggest LLE is generally sufficient in a relevant concentration range. Both parameters were specifically assessed at two concentration levels (i.e., 0.5 and 1.0 µg L^−1^) of OTA spiked into wine; these two concentration levels were specifically selected as 87% (27 out of 31) of wines above the limits-of-quantitation (LOQ) were found to contain OTA within this range (i.e., 0.5 to 2.0 µg L^−1^; Table 1). Accuracy, measured as a mean percent of recovery, was 83% and 99%, respectively, whereas precision (i.e., %RSD) was 15% and 18%, respectively, for the two spiked concentration levels. Furthermore, precision based on repeated measurement of selected wines (measured in the range of 1.0 to 2.2 µg L^−1^) was found to be much better (i.e., 0.3–9.0%).

**Figure 1:**
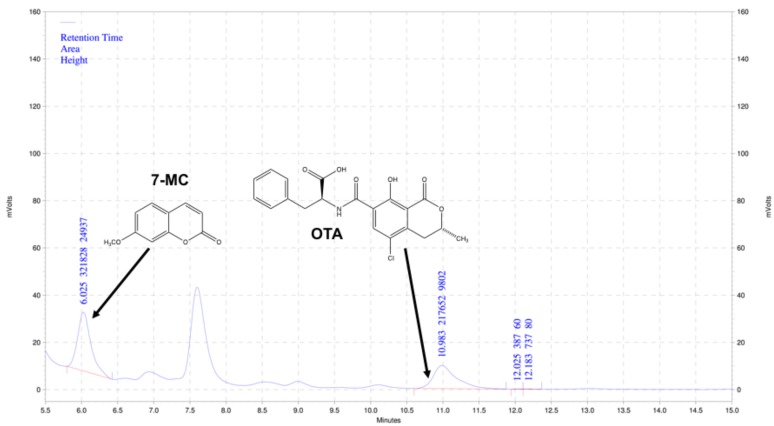
Chromatogram showing resolution of ochratoxin A (OTA) (2 µg L^−1^) and internal standard, 7-methoxycoumarin (100 µg L^−1^), by HPLC-FD.

Alongside the simplified LLE sample preparation step, the method developed in the present study adapted the readily available and relatively inexpensive 7-methoxycoumarin (7-MC), as a novel fluorescent internal standard. The internal standard and OTA were well resolved by HPLC-FD (Figure 1), and very high linearity (R^2^ = 0.9994) between peak ratios (i.e., OTA/7-MC) and OTA concentration, over a wide concentration range (0–90 µg L^−1^), was observed. Linearity is comparable with previous studies that utilized parabens for HPLC-FD (R^2^ = 0.9999 [49]), as well as HPLC-MS techniques employing the structurally unrelated mycotoxin, zearalenone (R^2^ > 0.999 [50]), and stable isotope dilution (R^2^ = 0.9981 [51]).

In terms of sensitivity, the limits of detection and quantitation (LOD and LOQ) of the analytical method were, on the other hand, relatively high (i.e., 0.1 and 0.3 µg L^−1^, respectively) compared to previous studies that typically report sensitivity on the order of parts-per-trillion [40]. The lower sensitivity is likely due, in part, to the relatively minimal sample preparation used in the present study. Most prior studies have utilized highly specific IAC cleanup, or other types of SPE, to remove constituents that might interfere with spectroscopic detection (i.e., HPLC-FD). The approach used in the present study, in contrast, employed simplified LLE, and subsequent sample concentration, as the only sample preparation step. Despite the lower sensitivity, however, the current method would be more than sufficient to meet the current (i.e., EU) regulatory guidelines of 2 µg L^−1^ OTA in wines. Taken together with good linearity, accuracy and precision, therefore, these results suggest that LLE coupled to HPLC-FD represents a sufficient method for rapid quantitative analysis of OTA in wine. Further supporting validation of the method, complementary analysis of samples by HPLC-MS/MS confirmed identity of OTA in both validation study samples, and subsequent assessment of U.S. wines (as discussed below).

### 2.2. Incidence and Measured Levels of OTA in U.S. Wines

Following validation of the LLE/HPLC-FD method, a sample (41 in total) of U.S. wines was evaluated for OTA. Wines included a range of varietals, and both red and white, as well as dry and sweet, representatives. The sample, furthermore, represented geographically diverse sources within the U.S. including growing regions in 8 states, although owing to a disproportionately large share of the market and, thus, available wines, the overall sample was heavily biased (>50%) toward wines sourced from California. California, in fact, includes four primarily recognized growing regions (namely the North Coast, Central Coast, South Coast and Central Valley) each of which is comparable in size to the growing regions of any of the other states. The current study included wines from three of these four growing regions in California (i.e., North Coast, Central Valley and Central Coast) and, therefore, 11 growing regions in total. Vintages of the wines spanned 2010 to 2015.

Both relatively high incidence, and measured levels, of OTA, were generally observed compared, in particular, to previous surveys of wines (Table 2; discussed below). Of the 41 wines evaluated by HPLC-FD (Table 1), more than 85% (i.e., 35 out of 41) were above the LOD (>0.1 µg L^−1^), and 31 of these OTA-positive wines (i.e., 89%) were above the LOQ (>0.3 µg L^−1^). In a complementary analysis, HPLC-MS/MS confirmed the identity of OTA in nearly three-quarters (i.e., 26 of the 35) of the wines positive (>LOD) by HPLC-FD, as well as 70% (22 out of 31) of the wines above LOQ. With respect to levels of OTA, the average concentration (for all wines) was approximately 1.3 µg L^−1^ with more than half (21 out of 41) of all wines—and 68% of wines above the LOQ (21 out of 31)—with levels greater than 1 µg L^−1^. Moreover, two wines had measured levels of OTA greater than the current EU regulatory limit (i.e., 2 µg L^−1^), and one was repeatedly measured to be remarkably high at approximately 8.6 ± 4.8 µg L^−1^. Presence of OTA was confirmed, in both cases, by HPLC-MS/MS.

Relevant characteristics of wines (i.e., dry versus sweet, red versus white) were considered with respect to presence and levels of OTA. Previous studies have reported higher levels of OTA in red wines [48,52,53,54]. Specifically, it has been argued that musts used to make red wine tend to have higher levels of OTA due to the extended period of contact between the skins and juice, as compared to white wines, during fermentation [54]. Similarly, previous studies have suggested that sweet wines are higher in OTA due to later harvest, and longer time spent on the vine, and thus, extended potential for exposure to OTA-producing fungal contaminants [23]. A higher number of red (54.8%) than white (40.0%) wines were found to contain OTA levels >1 µg L^−1^ in the present study (Table 1). However, this difference was not significant (Chi-Square test, *p* > 0.05), and averaged OTA concentrations for red versus white wines (Table 1) were, likewise, not significantly different. Interestingly, in contrast to previous studies, a higher number of dry (58.1%) versus sweet (30%) wines were found (Table 1) to contain these higher levels (>1 µg L^−1^) of OTA, although the difference was also not statistically significant (Chi-Square test, *p* > 0.05). This observed difference between dry and sweet, in the present study, may be confounded by a highly disproportionate number of dry red (29 of 32) versus dry white (3 of 9) wines such that this difference may simply reflect relatively higher levels of OTA in red wines (and, more generally, lower number of sweet wines) in the current study. Indeed, one of the two wines above the 2 µg L^−1^ regulatory guideline—and in fact, the highest measured OTA concentration (Table 1)—was a sweet, white wine.

Prevalence and levels of OTA observed in the current study are generally higher than reported in previous surveys including assessments of U.S. wines. Notably, the present study is the first in more than a decade to survey OTA in U.S. wines. In a global survey, Soleas et al. (2001) [14] reported only 11% of U.S. red wines with OTA concentrations above a reporting limit of 0.05 µg L^−1^; none of the white wines assayed were positive for OTA. Similarly, as part of a broader comparative assessment between Canadian and various international wines [19], OTA was detected in only 8.7% of the U.S. representatives, and moreover, none were above the LOQ (0.050 µg L^−1^) of the method used (i.e., HPLC-FD coupled to IAC). The sample, in this previous report, however, was relatively smaller (i.e., 23 wines), and regional diversity was not reported. In the only other subsequent study [16] to specifically assess U.S. wines (including 84 from 9 states), on the other hand, OTA was quantifiable in approximately 31% of the wines with concentrations as high as 1.68 µg L^−1^. The mean concentration of OTA in this 2003 study, however, was nearly two-orders of magnitude lower (i.e., 0.044 µg L^−1^ for red wines, 0.010 µg L^−1^ for white wines) than present study results (~1.3 µg L^−1^).

Compared to numerous studies of internationally (i.e., non-U.S.) sourced wines, and particularly those analyzed by similar (i.e., HPLC-FD) methods, both frequency and levels of OTA in the current study, likewise, rank amongst the highest reported (Table 2). Prevalence and measured levels of OTA among U.S. wines in the present study are seemingly comparable, however, to similar previous surveys of wines from several climatically warmer Southern European and Mediterranean sources which are, in particular, associated with the so-called “Mediterranean” viticultural classification of climate (loosely based on the *Köppen–Geiger climate classification* system [55,56]). The previously highest reported incidences and/or measured levels of OTA have, indeed, been found among wines from Italy, Spain, Portugal, Greece, Turkey, Morocco and Tunisia [12,13,17,18,21,23,24,25,30,32,33,37,48]. And OTA levels exceeding the EU limit (i.e., 2 µg L^−1^) have been regularly reported for wines from each of these countries—with the exception of Tunisia for which a maximum of 1.50 µg L^−1^ OTA has been reported [37]—at frequencies ranging in frequencies from ≤1% to more than 10% of the wines evaluated (Table 2). With very few exceptions, on the other hand, wines in excess of the 2 µg L^−1^ limit have not been reported from previous surveys spanning geographically diverse regions including Northern and Central Europe (e.g., France, Croatia, Slovakia, Moravia, [22,28,29,35]), Canada [19], South America (e.g., Chile, Argentina, Brazil [20,31,36,38]), Asia (e.g., China [34]) and South Africa [15] which are, on the other hand, largely associated with either more climatologically drastic *continental*, or moderated *maritime* (i.e., oceanic), viticultural climate classifications.

**Figure 2:**
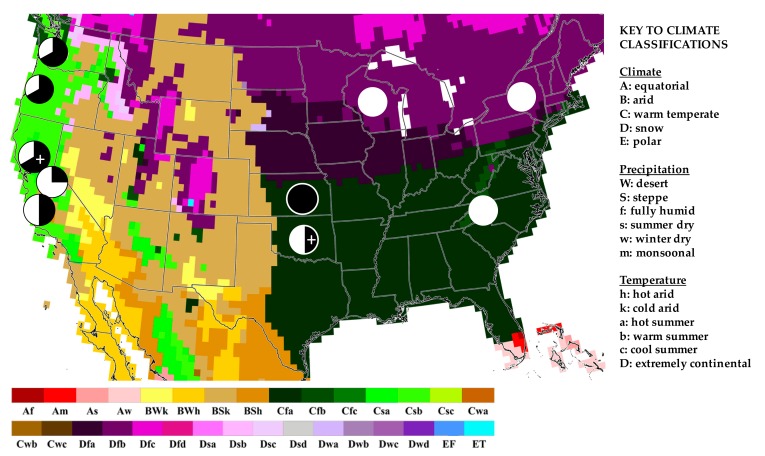
Incidence of high OTA levels of wines overlaid on Köppen–Geiger climate classifications of the U.S. Prevalence of wines with measured levels of OTA greater than 1 µg L^−1^ are shown by pie-charts: Black indicates percent of wines >1 µg L^−1^. Wines above the current EU regulatory limit (2 ppb) are denoted by +. Reproduced from [56], 2006, Schweizerbart science publishers.

Further studies and, in particular, a larger and more inclusive sample are clearly needed, but a regional assessment suggests a possible pattern with respect to the geographical source of the U.S. wines in relation to OTA (Figure 2). High levels of OTA (e.g., >1 µg L^−1^) were restricted to wines from Pacific coastal (i.e., California, Oregon and Washington) and warmer, more southerly Midwest (i.e., Oklahoma, Kansas) growing regions (Figure 2) which are classified (in the Köppen–Geiger climate classification system) as either Mediterranean or sub-tropical, respectively. Levels of OTA in excess of 1 µg L^−1^ are, however, absent from wines sourced from more “continental” climates (i.e., New York, Wisconsin, North Carolina highlands). The same trend is more generally reflected in the regional differences between measured levels of OTA in wines (Table 3).

Notably, this pattern seems to mirror international patterns, as discussed above, whereby higher levels of OTA are largely associated with wines from regions classified among the Mediterranean viticultural classification, or otherwise consistently warmer climates, compared with continental sources (Table 2). A similar observation has, indeed, been previously reported in multiple surveys of European wines. Multiple studies [13,24,25] have, for example, documented higher levels of OTA among wines from southern Mediterranean, compared to northern continental, regions of Italy. In addition, a more general trend with respect to southern and northern regions of Europe has been noted [57,58], although a more recent study of Mediterranean wines has questioned this trend [59]. Similarly, previous studies in both Italy and Greece have also noted higher occurrence and levels of OTA among wines from coastal (i.e., island) regions, and have attributed this to climatic conditions including higher moisture due, in particular, to seasonal winds from, in these cases, the Mediterranean Sea [17,25]. Whether geographic variability in the current survey might be related to local growing climate, or related environmental factors, or to varietals grown in these regions (or other factors), remains to be investigated in future studies.

## 3. Conclusions

The current study demonstrated a simplified HPLC-FD method, specifically coupled to LLE, to be both rapid and sufficiently quantitative for assessment of OTA in wine. And subsequent assessment of U.S. wines revealed a relatively high incidence, and measured levels, of OTA compared to previous surveys. The factors which contribute to the occurrence of OTA in wine, including those related to harvesting practices (e.g., sweet versus dry) and to the wine-making process (i.e., red versus white), as well as growing conditions, remain to be clarified. Emerging evidence, however, suggests an effect of climate and, thus, climate change including both long term (e.g., decadal) rises, and even sporadic (e.g., single year) increases, in temperature and other related variables (e.g., humidity/moisture) to the occurrence of mycotoxins in agricultural products [60,61,62]. Apparent geographic patterns observed in the present study, and in particular, higher levels of OTA in wines from warmer “Mediterranean”-like and sub-tropical climates (Figure 2 and Table 3), would align with a role of climate in the occurrence of OTA in wine. Moreover, the generally high incidence and measured levels of OTA observed in the current survey—as the first in more than a decade—would be consistent with a possible role of climate change on a decadal scale. Future studies, to investigate this proposed role of climate, are clearly needed. More generally, however, the current study reinforces the need for regular surveillance of OTA in wine, and other agricultural products, as well as investigation of factors which drive OTA contamination.

## 4. Materials and Methods

### 4.1. Wine Samples and Chemicals

A representative sample of U.S. wines was obtained through donation from the Chaplin School of Hospitality and Tourism Management at FIU. In total, 41 wines were analyzed, including 31 red (28 dry and 3 sweet) and 10 white (3 dry and 7 sweet) wines. Wines represented 10 growing regions from 8 states (including 3 of the 4 primary growing regions in California). No regional information was available for two wines. Vintages of the wines spanned 2010 to 2015. Samples were transferred by pipetting in the laboratory from bottles to 15-mL polypropylene tubes, and stored at −20 °C until analysis. All analyses were done between May and August 2015.

Chemicals and reagents were purchased from several vendors: OTA standard was purchased from Enzo Life Sciences (Farmingdale, NY, USA); all solvents including chloroform, acetonitrile and methanol (MeOH) used were LC-MS grade (OmniSolv^®^, VWR Scientific, Radnor, PA, USA); and 7-methoxycoumarin (7-MC) was purchased from Sigma-Aldrich (St. Louis, MO, USA).

### 4.2. Extraction and Sample Preparation for HPLC Analysis

For HPLC-FD and HPLC-MS/MS analyses, wine samples were extracted by a liquid/liquid extraction (LLE) method. Briefly, to a 1.5-mL Eppendorf tube containing a 500 µL aliquot of each wine sample, 100 µL of internal standard (i.e., 100 µg L^−1^ 7-MC) and 1 mL of chloroform were added. The sample was vortex-mixed for 10 s, and allowed to equilibrate for 10 min. The aqueous layer was removed by pipetting, and the chloroform layer was dried under a stream of air. The residue was subsequently reconstituted with 200 µL of HPLC mobile phase (consisting of water, acetonitrile and 2% glacial acetic acid (49.5:49.5:1)), and transferred into HPLC vials (containing 200-µL inserts) for subsequent analyses.

### 4.3. HPLC-FD of OTA in Wines

Following extraction and subsequent sample preparation, OTA from wine was evaluated in triplicate (*n* = 3) by HPLC-FD using a method adapted from Visconti et al. [48], and a Shimadzu Prominence UFLC system equipped with autosampler and fluorescence detector. The injection volume was 20 µL per sample, and separation was accomplished using Sonoma C18 (2) analytical column (5 µm, 100 Å pore size, 250 mm × 4.6 mm; ES Industries, West Berlin, NJ, USA) by isocratic elution (49.5:49.5:1 water/acetonitrile/2% glacial acetic acid) at 1.0 mL/min. Column oven temperature was kept at 30 °C. Fluorescence detector wavelengths for OTA (and internal standard) were 333 nm excitation and 460 nm emission. Peak integration was done with Shimadzu EZStart software (Ver. 7.4, Shimadzu Scientific Instruments, Inc., Canby, OR, USA). Quantitation of OTA was based on commercially available OTA standards, and internal standard, i.e., 7-MC, spiked into a red wine, previously determined to contain no toxin, which was extracted alongside samples. Ochratoxin A and the internal standard were detected at retention times 10.9 and 6.0 min, respectively (Figure 1), and concentration was correlated to relative peak area (i.e., OTA/MC-7) by the method of least squares.

### 4.4. Analytical Performance of HPLC-FD

As the primary analytical method, HPLC-FD coupled to LLE, and specifically using 7-MC as internal standard, was validated for OTA over a range of <LOQ to 90 µg L^−1^. Linearity was assessed by method of least squares, and associated correlation coefficient (R^2^), based on linear regression (i.e., calibration curve) between relative peak area of OTA/7-MC and OTA concentration. Sensitivity was evaluated in terms of limits of detection (LOD) and limits of quantitation (LOQ), and calculated as 3 s/m and 10 s/m, respectively, where *s* is the standard deviation from repeated (*n* = 4) measurement of a blank sample, and *m* is the slope of the calibration curve (between relative peak area of OTA/7-MC and OTA concentration). Recovery, accuracy and precision were assessed for two relevant concentration levels (0.5 and 1.0 µg L^−1^) of OTA spiked into a red wine previously confirmed to contain no detectable OTA. Accuracy was calculated as percent recovery, and associated error, from the two concentration levels (*n* = 6). Precision was determined as percent relative standard deviation (%RSD) for repeated measurements (*n* = 6). Uncertainty (%RSD) was additionally assessed by repeated measurement (*n* = 3) of wine samples.

### 4.5. HPLC-MS/MS of OTA in Wines

In parallel with HPLC-FD, OTA extracted from wine (by LLE) was analyzed by HPLC-MS/MS as a confirmatory method. Analyses were conducted using Thermo Scientific TSQ Quantum Access instrument equipped with Accela HPLC. The injection volume was 20 µL per sample (via autosampling system), and analytical separation was achieved using a Kinetex^®^ C18 column (2.6 µm, 100 Å, 50 × 2.1 mm) with isocratic elution by 49.5:49.5:1 water/acetonitrile/2% acetic acid with flow rate of 350 µL min^−1^. The ion source settings were as follows: discharge current of 4.0, vaporization temperature of 150 °C, sheath gas pressure of 30 units, ion sweep gas pressure of 0 units, auxilliary gas pressure of 20 units, capillary temperature of 300 °C, capillary offset of 35 units, tube lens offset of 93 units, skimmer offset of −7 units. Selected Reaction Monitoring (SRM) was used for MS/MS analysis; OTA was monitored in negative ionization mode, where the parent ion was *m*/*z* 402, and the daughter ions were *m*/*z* 358 and 314.

## Figures and Tables

**Table 1:** Prevalence and measured concentrations (µg L^−1^) of OTA among U.S. wines.

Type (# Wines)	>LOD (%)	Mean	Median	Range	^1^ Number (%) of Wines in the Concentration Range, µg L^−1^					
					<0.3	0.3–0.5	0.5–1.0	>1.0 ^2^	>2.0 ^3^	>5.0
**Red (31)**	**28 (90.3)**	**1.0**	**1.0**	**0.3–2.1**	**3 (9.7)**	**2 (6.5)**	**6 (19.4)**	**17 (54.8)**	**1 (3.2)**	**0 (0.0)**
Sweet (3)	2 (66.7)	0.8	0.7	0.5–1.1	0 (0.0)	0 (0.0)	1 (33.3)	1 (33.3)	0 (0.0)	0 (0.0)
Dry (28)	26 (92.9)	1.0	1.1	0.3–2.1	3 (10.7)	2 (7.1)	5 (17.9)	16 (57.1)	0 (0)	0 (0.0)
**White (10)**	**7 (70.0)**	**2.6**	**1.2**	**0.6–8.6**	**1 (10.0)**	**0 (0.0)**	**2 (20.0)**	**4 (40.0)**	**1 (10.0)**	**1 (10.0)**
Sweet (7)	4 (57.1)	5.0	5.0	1.4–8.6	1 (14.3)	0 (0.0)	1 (14.3)	2 (28.6)	1 (14.3)	1 (14.3)
Dry (3)	3 (100.0)	0.9	1.1	0.6–1.2	0 (0.0)	0 (0.0)	1 (33.3)	2 (66.7)	0 (0.0)	0 (0.0)
**Sweet (10)**	**6 (60.0)**	**2.5**	**1.1**	**0.5–8.6**	**1 (10.0)**	**0 (0.0)**	**2 (20.0)**	**3 (30.0)**	**1 (10.0)**	**1 (0.0)**
**Dry (31)**	**29 (93.5)**	**1.0**	**1.1**	**0.3–2.1**	**3 (9.7)**	**2 (6.5)**	**6 (19.4)**	**18 (58.1)**	**1 (3.2)**	**0 (0.0)**
**Total (41)**	**35 (85.4)**	**1.3**	**1.1**	**0.3–8.6**	**4 (9.8)**	**2 (4.9)**	**8 (19.5)**	**21 (51.2)**	**2 (4.9)**	**1 (2.4)**

^1^ Categories include only samples above LOD. Percent of wines (given in parentheses) represent number within each range divided by total number of wines of each “Type”. Samples in the “<0.3” µg L^−1^ category are below LOQ. ^2^ Category includes samples counted in >2.0 and >5.0 categories. ^3^ Category includes samples counted in >5.0 category.

**Table 2:** Comparison of OTA measured in the present study to previous studies.

Region	Range (µg L^−1^)	>2 µg L^−1^ # Wines (%)	Analytical Method (Sample Preparation)	Reference
**North America**				
U.S.A.	0.3–8.6	2/41 (4.9%)	HPLC-FD (LLE)	Present study
	0.01–1.68	-	HPLC-FD (IAC)	[16]
Canada	0.050–0.393	-	HPLC-FD (IAC)	[19]
**Europe**				
Italy	0.2–3.177	N/A	HPLC-FD (IAC)	[13]
	0.1–7.63	6/55 (10.9%)	HPLC-FD (IAC)	[48]
	0.2–4.93	9/112 (8.0%)	HPLC-FD (IAC)	[24]
	0.03–7.50	22/783 (2.8%)	HPLC-FD (IAC)	[25]
	0.01–2.63	29/1206 (2.4%)	HPLC-FD (IAC)	[32]
Greece	0.05–2.00	1/105 (<1.0%)	HPLC-FD (IAC)	[33]
	0.05–2.69	N/A	HPLC-FD (IAC)	[18]
	0.02–3.20	3/35 (8.6%)	HPLC-FD (IAC)	[17]
Spain	0.02–4.63	18/188 (9.6%)	HPLC-FD (IAC)	[23]
Croatia	0.014–0.021	-	HPLC-FD (IAC)	[28]
Turkey	0.01–2.3	1/47 (2.1%)	HPLC-FD (LLE/SPE)	[21]
				[27]
Slovakia	0.036–0.463	-	HPLC-FD (IAC)	[22]
Moravia	0.001–0.072	-	HPLC-FD (IAC)	[35]
Portugal	1.23–2.4	1/60 (1.7%)	HPLC-FD (direct inject)	[30]
France	0.31–0.92	-	HPLC-FD (IAC)	[29]
**South America**			HPLC-FD (IAC)	
Argentina	0.028–0.042	-	HPLC-FD (IAC)	[20]
	2.00–4.82	3/47 (6.4%)	HPLC-FD (IAC)	[31]
	0.05–0.98	-	HPLC-MS/MS (SPE)	[38]
Chile	0.028–0.071	-	HPLC-FD (IAC)	[20]
	0.14–0.35	-	HPLC-FD (IAC)	[36]
Brazil	0.028–0.042	-	HPLC-FD (IAC)	[20]
**Africa**				
South Africa	0.04–0.39	-	HPLC-FD (IAC)	[15]
Tunisia	0.09–1.50	-	HPLC-FD (IAC)	[37]
Morocco	0.028–3.24	1/30 (3.3%)	HPLC-FD (SPE)	[12]
**Asia**				
China	0.09–1.18	-	HPLC-FD (IAC)	[34]

**Table 3:** Levels of OTA in U.S. wines by growing region.

State (# Wines) ^1^	Region (# Wines)	>LOQ (%)	Average [OTA], µg L^−1^	Range ^2^, µg L^−1^
CA (24)		21 (87.5%)	1.0	0.3–2.1
	Central Coast (2)	2 (100.0%)	0.9	0.7–1.1
	Central Valley (4)	3 (75.0%)	1.0	0.6–1.4
	North Coast (18)	16 (88.9%)	1.0	0.3–2.1
KS (1)		1 (100.0%)	1.4	≤1.4
NC (1)		1 (100.0%)	1.0	≤1.0
NY (3)		1 (33.3%)	0.5	≤0.5
OK (2)		1 (50%)	8.6	≤8.6
OR (3)		2 (66.7%)	1.4	1.3–1.5
WA (3)		3 (100%)	0.9	0.6–1.1
WI (2)		0 (0%)	<LOQ	N/A

^1^ State abbreviations: CA = California; KS = Kansas; NC = North Carolina; NY = New York; OK = Oklahoma; OR = Oregon; WA = Washington; WI = Wisconsin; ^2^ For regions with only one wine above the LOQ, only this measured concentration (as maximum concentration) is given. For regions with no wines above LOQ, no concentration range is available (N/A).

## References

[1] Van Egmond H.P., Speijers G.J.A. (1994). Survey of data on the incidence and levels of ochratoxin A in food and animal feed worldwide. J. Nat. Toxins.

[2] Bennet J.W., Klich M. (2003). Mycotoxins. Clin. Microbiol. Rev..

[3] Heussner A.H., Bingle L.E.H. (2015). Comparative ochratoxin toxicity: A review of the available data. Toxins.

[4] Haq M., Gonzalez N., Mintz K., Jaja-Chimedza A., De Jesus C.L., Lydon C., Welch A.Z., Berry J.P. (2016). Teratogenicity of ochratoxin A, and the degradation product, ochratoxin α, in the zebrafish (*Danio rerio*) embryo model of vertebrate development. Toxins.

[5] Malir F., Ostry V., Pfohl-Leszkowicz A., Malir J., Toman J. (2016). Ochratoxin A: 50 years of research. Toxins.

[6] Duarte S.C., Lino C.M., Pena A. (2010). Mycotoxin food and feed regulation and the specific case of ochratoxin A: A review of the worldwide status. Food Addit. Contam. Part A.

[7] International Wine and Spirit Record (IWSR) (2012). Global Wine Consumption Set to Increase by 2 Billion Bottles. http://www.westernfarmpress.com/grapes/global-wine-consumption-set-increase-2-billion-bottles.

[8] McMillan R. (2017). State of the Wine Industry. Silicon Valley Bank Wine Division. https://www.svb.com/uploadedFiles/Content/Trends_and_Insights/Reports/Wine_Report/2017-wine-report.pdf.

[9] Wine Institute. https://www.wineinstitute.org/resources/statistics/article86.

[10] Abrunhosa L., Paterson R.R.M., Venancio A. (2010). Biodegradation of ochratoxin A for food and feed decontamination. Toxins.

[11] Esti M., Benucci I., Liburdi K., Acciaro G. (2012). Monitoring of ochratoxin A fate during alcoholic fermentation of wine-must. Food Control.

[12] Filali A., Ouammi L., Betbeder A.M., Baudrimont I., Soulaymani R., Benayada A., Creppy E.E. (2001). Ochratoxin A in beverages from Morocco: A preliminary survey. Food Addit. Contam..

[13] Pietri A., Bertuzzi T., Pallaroni L., Piva G. (2001). Occurrence of ochratoxin A in Italian wines. Food Addit. Contam..

[14] Soleas G.J., Yan J., Goldberg D.M. (2001). Assay of ochratoxin A in wine and beer by high-pressure liquid chromatography photodiode array and gas chromatography mass selective detection. J. Agric. Food Chem..

[15] Shephard G.S., Fabiani A., Sotckenström S., Mschicileli N., Sewram V. (2003). Quantitation of ochratoxin A in South African wines. J. Agric. Food Chem..

[16] Siantar D., Halverson C.A., Kirmiz C., Peterson G.F., Hill N.R., Dugar S.M. (2003). Ochratoxin A in wine: Survey by antibody- and polymeric-based SPE columns using HPLC/fluorescence detection. Am. J. Enol. Vitic..

[17] Soufleros E.H., Tricard C., Bouloumpasi E. (2003). Occurrence of ochratoxin A in Greek wines. J. Sci. Food Agric..

[18] Stefanaki I., Foufa E., Tsatsou-Dritsa A., Dais P. (2003). Ochratoxin A concentrations in Greek domestic wines and dried vine fruits. Food Addit. Contam..

[19] Ng W., Mankotia M., Pantazopoulos P., Neil R.J., Scott P.M. (2004). Ochratoxin A in wine and grape juice sold in Canada. Food Addit. Contam..

[20] Rosa C.A.R., Magnoli C.E., Fraga M.E., Dalcero A.M., Santana D.M.N. (2004). Occurrence of ochratoxin A in wine and grape juice marketed in Rio de Janeiro, Brazil. Food Addit. Contam..

[21] Anli E., Cabuk B., Vural N., Baspinar E. (2005). Ochratoxin in Turkish wines. J. Food Biochem..

[22] Belajova E., Rauova D. (2007). Determination of ochratoxin A and its occurrence in wines of Slovakian retail. J. Food Nutr. Res..

[23] Burdaspal P., Legarda T. (2007). Occurrence of ochratoxin A in sweet wine produced in Spain and other countries. Food Addit. Contam..

[24] Perrone G., Nicoletti I., Pascale M., De Rossi A., De Girolamo A., Visconti A. (2007). Positive correlation between high levels of ochratoxin A and resveratrol-related compounds in red wines. J. Agric. Food Chem..

[25] Brera C., Debegnach F., Minardi V., Prantera E., Pannunzi E., Faleo S., de Santis B., Miraglia M. (2008). Ochratoxin A contamination in Italian wine samples and evaluation of the exposure in the Italian population. J. Agric. Food Chem..

[26] Valero A., Marín S., Ramos A.J., Sanchis V. (2008). Survey: Ochratoxin A in European special wines. Food Chem..

[27] Altiokka G., Can N.O., Atkosar Z., Aboul-Enein H.Y. (2009). Determination of ochratoxin A in Turkish wines. J. Food Drug Anal..

[28] Flajs D., Domijan A.M., Ivic D., Cvjetkovic B., Peraica M. (2009). ELISA and HPLC analysis of ochratoxin A in red wines of Croatia. Food Control.

[29] Radoi A., Dumitru L., Barthelmebs L., Marty J.L. (2009). Ochratoxin A in some French wines: Application of a dirct competitive ELISA based on an OTA-HRP conjugate. Anal. Lett..

[30] Pena A., Cerejo F., Silva L.J., Lino C.M. (2010). Ochratoxin A survey in Portugese wine by LC-FD with direct injection. Talanta.

[31] Ponsone M.L., Chiotta M.L., Combina M., Torres A., Knass P., Dalcero A., Chulze S. (2010). Natural occurrence of ochratoxin A in musts, wines and grape vine fruits from grapes harvested in Argentina. Toxins.

[32] Spadaro D., Loré A., Garibaldi A., Gullino M.L. (2010). Occurrence of ochratoxin A before bottling in DOC and DOCG wines produced in Piedmont (Northern Italy). Food Control.

[33] Labrinea E.P., Natskoulis P.I., Spiropoulos A.E., Magan N., Tassou C.C. (2011). A survey of ochratoxin A occurrence in Greek wines. Food Addit. Contam. Part B Surveill..

[34] Wu J., Tan Y., Wang Y., Xu R. (2011). Occurrence of ochratoxin A in wine and beer samples from China. Food Addit. Contam. Part B Surveill..

[35] Mikulikova R., Belakova S., Benesova K., Svoboda Z. (2012). Study of ochratoxin A content in South Moravian wines by the UPLC method with fluorescence detection. Food Chem..

[36] Vega M., Rios G., von Baer D., Mardones C., Tessini C., Herlitz E., Saelzer R., Ruiz M.A. (2012). Ochratoxin A occurrence in wines produced in Chile. Food Control.

[37] Lasram S., Oueslati S., Chebil S., Mliki A., Ghorbel A. (2013). Occurrence of ochratoxin A in domestic beer and wines from Tunisia by immunoaffinity clean-up and liquid chromatography. Food Addit. Contam. Part B Surveill..

[38] Mariño-Repizo L., Gargantini R., Manzano H., Raba J., Cerutti S. (2016). Assessment of ochratoxin A occurrence in Argentine red wines using a novel sensitive QuEChERS-solid phase extraction approach prior to ultra high performance liquid chromatography-tandem mass spectrometry methodology. J. Sci. Food Agric..

[39] Mira de Orduña R. (2010). Climate change associated effects on grape and wine quality and production. Food Res. Int..

[40] Skarkova J., Ostry V., Malir F., Roubal T. (2013). Determination of ochratoxin A in food by high performance liquid chromatography. Anal. Lett..

[41] Al-Taher F., Banaszewski K., Jackson L., Zweigenbaum J., Ryu D., Cappozzo J. (2013). Rapid method for the determination of multiple mycotoxins in wines and beers by LC-MS/MS using a stable isotope dilution assay. J. Agric. Food Chem..

[42] Li P., Zhang Z., Hu X., Zhang Q. (2013). Advanced hyphenated chromatographic-mass spectrometry in mycotoxin determination: Current status and prospects. Mass Spectrom. Rev..

[43] Rizzazzi-Fazeli E., Reiter E.V., De Saeger S. (2011). Sample preparation and clean up strategies in the mycotoxin analysis: Principles, applications and recent developments. Determining Mycotoxins and Mycotoxigenic Fungi in Food and Feed.

[44] Fabiani A., Corzani C., Arfelli G. (2010). Correlation between different clean-up methods and analytical techniques performances to detect ochratoxin A in wine. Talanta.

[45] Huertas-Perez J.F., Arroyo-Manzanares N., Garcia-Campaña A.M., Gamiz-Gracia L. (2016). Solid-phase extraction as sample treatment for the determination of ochratoxin A in foods: A review. Crit. Rev. Food Sci. Nutr..

[46] Arroyo-Manzares N., Garcia-Campaña A.M., Gamiz-Gracia L. (2011). Comparison of different sample treatments for the analysis of ochratoxin A in wine by capillary HPLC with laser-induced fluorescence detection. Anal. Bioanal. Chem..

[47] Pizzutti I.R., de Kok A., Scholten J., Righi L.W., Cardoso C.D., Rohers G.N., da Silva R.C. (2014). Development, optimization and validation of a multimethod for the determination of 36 mycotoxins in wines by liquid chromatography-tandem mass spectrometry. Talanta.

[48] Visconti A., Pascale M., Centonze G. (1999). Determination of ochratoxin A in wine by means of immunoaffinity column clean-up and high-performance liquid chromatography. J. Chromatogr. A.

[49] Tunçel M., Öncü Kaya E.M., Uysal U.D., Güray T. (2015). HPLC fluorescence determination of ochratoxin A utilizing double internal standard and its application to poultry feed. Turk. J. Chem..

[50] Leitner A., Zöllner P., Paolillo A., Stroka J., Papadopoulou-Bouraoui A., Jaborek S. (2002). Comparison of methods for determination of ochratoxin A in wine. Anal. Chim. Acta.

[51] Roland A., Bros P., Bouisseau A., Cavelier F., Schneider R. (2014). Analysis of ochratoxin A in grapes, musts and wines by LC-MS/MS: First comparison of stable isotope dilution assay and diastereomeric dilution assay methods. Anal. Chim. Acta.

[52] Battiliani P., Magan N., Logrieco A. (2006). European research on ochratoxin A in grapes and wine. Int. J. Food Microbiol..

[53] Mateo R., Medina A., Mateo E.M., Mateo F., Jimenez M. (2007). An overview of ochratoxin A in beer and wine. Int. J. Food Microbiol..

[54] Visconti A., Perrone G., Cozzi G., Solfrizzo M. (2008). Managing ochratoxin A risk in the grape-wine food chain. Food Addit. Contam. A.

[55] Spellman G. (1999). Wine, weather and climate. Weather.

[56] Kottek M., Grieser J., Beck C., Rudolf B., Rubel F. (2006). World map of the Köppen-Geiger climate classification updated. Meteorol. Z..

[57] Zimmerli B., Dick R. (1996). Ochratoxin A in table wine and grape-juice: Occurrence and risk assessment. Food Addit. Contam..

[58] Ottender H., Majerus P. (2000). Occurrence of ochratoxin A (OTA) in wines: Influences of the type of wine and its geographical origin. Food Addit. Contam..

[59] Remiro R., Irigoyen A., González-Peñas E., Lizarraga E., López de Cerain A. (2013). Levels of ochratoxins A in Mediterranean red wines. Food Control.

[60] Kos J., Mastilovic J., Hajinal E.J., Saric B. (2013). Natural occurrence of aflatoxins in maize harvested in Serbia during 2009–2012. Food Control.

[61] Dobolyi C., Sebok F., Varga J., Kocsube S., Szigeti G., Baranyi N., Szecsi A., Toth B., Varga M., Kriszt B. (2013). Occurrence of aflatoxin producing *Aspergillus flavus* isolates in maize kernel in Hungary. Acta Aliment..

[62] Paterson R.R.M., Lima N. (2017). Thermophilic fungi dominate aflatoxigenic/mycotoxigenic fungi on food under global warming. Int. J. Environ. Res. Public Health.

